# Attracting, trapping and killing disease-transmitting mosquitoes using odor-baited stations - The Ifakara Odor-Baited Stations

**DOI:** 10.1186/1756-3305-3-12

**Published:** 2010-03-01

**Authors:** Fredros O Okumu, Edith P Madumla, Alex N John, Dickson W Lwetoijera, Robert D Sumaye

**Affiliations:** 1 Biomedical and Environmental Sciences Thematic Group, Ifakara Health Institute, PO Box 53, Ifakara, Tanzania; 2 Disease Control and Vector Biology Unit, London School of Hygiene and Tropical Medicine, Keppel Street, WCIE 7HT, London UK; 3 Department of Zoology and Wildlife Management, University of Dar es Salaam, PO Box 35091, Dar es Salaam, Tanzania

## Abstract

**Background:**

To accelerate efforts towards control and possibly elimination of mosquito-borne diseases such as malaria and lymphatic filariasis, optimally located outdoor interventions could be used to complement existing intradomicilliary vector control methods such as house spraying with insecticides and insecticidal bednets.

**Methods:**

We describe a new odor-baited station for trapping, contaminating and killing disease-transmitting mosquitoes. This device, named the 'Ifakara Odor-baited Station' (Ifakara OBS), is a 4 m^3 ^hut-shaped canvas box with seven openings, two of which may be fitted with interception traps to catch exiting mosquitoes. It is baited with synthetic human odors and may be augmented with contaminants including toxic insecticides or biological agents.

**Results:**

In field trials where panels of fabric were soaked in 1% pirimiphos-methyl solution and suspended inside the Ifakara OBS, at least 73.6% of *Anopheles arabiensis*, 78.7% of *Culex *and 60% of *Mansonia *mosquitoes sampled while exiting the OBS, died within 24 hours. When used simply as a trap and evaluated against two existing outdoor traps, Ifakara Tent trap and Mosquito Magnet-X^®^, the OBS proved more efficacious than the Ifakara Tent trap in catching all mosquito species found (P < 0.001). Compared to the Mosquito Magnet-X^®^, it was equally efficacious in catching *An. arabiensis *(P = 0.969), but was less efficacious against *Culex *(P < 0.001) or *Mansonia *species (P < 0.001).

**Conclusion:**

The Ifakara OBS is efficacious against disease-carrying mosquitoes including the malaria vector, *An. arabiensis *and Culicine vectors of filarial worms and arboviruses. It can be used simultaneously as a trap and as a contamination or killing station, meaning most mosquitoes which escape trapping would leave when already contaminated and die shortly afterwards. This technique has potential to complement current vector control methods, by targeting mosquitoes in places other than human dwellings, but its effectiveness in the field will require cheap, long-lasting and easy-to-use mosquito lures.

## Introduction

Development and adoption of alternative mosquito control tools has been exceptionally slow over the past several years. As such, existing intradomicilliary methods, namely indoor residual insecticide spraying (IRS) and insecticide treated nets (ITNs) have remained the primary interventions against vectors of important pathogens such as those that cause malaria and dengue fever [1,2]. These methods have been considerably effective, for example when used alongside appropriate therapeutic measures, they have contributed to massive declines in malaria related morbidity and mortality in Africa [2-6].

Considering malaria as an example, the current global action plan focuses on achieving universal protective coverage with ITNs and IRS alongside diagnosis and treatment, but also on country by country elimination of malaria transmission [7]. Since 2007, there have also been calls for concerted efforts towards global malaria eradication [8,9]. Despite these developments, there is growing concern that existing tools may not be adequate to achieve these goals; and that alternative or complementary interventions are urgently needed [9-12].

Other than the fact that ITNs and IRS are insecticide-based and may be affected by physiological and behavioural resistance among mosquito populations [13-15], another limitation is that these methods target only mosquitoes that enter or those that attempt to enter human dwellings. Such strategies neglect the natural distribution of mosquitoes over geographical landscapes and may not achieve elimination especially in areas where vectors rest and bite outdoors [14-16], away from dwellings or where the vectors can survive on non human hosts found away from natural human aggregations [17,18]. While significant household protection can be achieved using existing vector control methods, communal protection and local elimination will require that transmission is targeted at more focal points than merely at household level.

This paper describes the development and field evaluation of an odor-baited station (OBS) that can be used to target host seeking mosquitoes in places other than human dwellings while considering geographical distributions of both mosquito and human populations. The device exploits a trade-off between benefits of luring mosquitoes and trapping them versus benefits of luring, contaminating and freeing the mosquitoes. By maximizing spaces through which mosquitoes can enter without being intercepted by any trapping device, and using a minimum number of efficient interception traps on a section of these open areas, the OBS acts both as a trap and as a contamination site so that mosquitoes which escape the trapping mechanism leave the device already contaminated and die shortly afterwards. This device, named the Ifakara OBS, is currently baited with a highly attractive synthetic lure recently constituted at the Ifakara Health Institute, Tanzania, and which was demonstrated to be 3 to 5 times more attractive than humans to various species of human biting mosquitoes [19]. The work represents the first attempt to develop a strategy to utilise this or similar chemical lures for future vector control.

## Methods

### The design of the Ifakara Odor-baited Station

**Figure 1:**
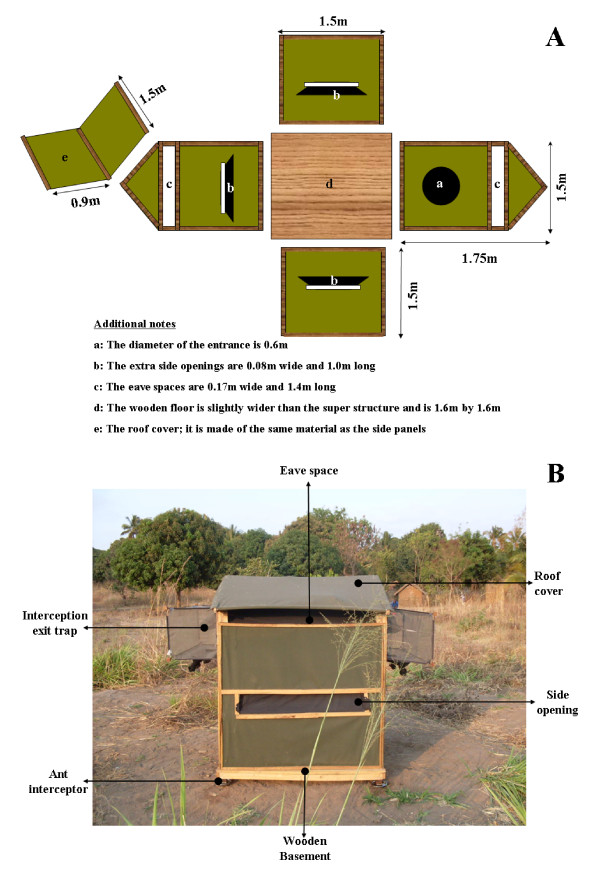
**The Ifakara Odor-Baited Station**. Panel **A**): A complete plan of the Ifakara OBS showing the dimensions and positions of mosquito entry and exit points. Panel **B**): A picture of the Ifakara OBS in-use, illustrating important features. The user entry point (not shown in this diagram) is located under the interception exit trap on the right side of the OBS.

The Ifakara OBS is essentially a hut-shaped box made of canvas on a wooden framework (Figs. 1A and 1B). It measures 1.5 m × 1.5 m and its highest point is 1.75 m from its wooden basement. On one side, it has a round operator entry point (0.6 m diameter) fitted with a black cotton sleeve. This sleeve prevents mosquitoes from exiting through the access whenever the operator is entering the OBS. In addition, the OBS has seven openings for mosquito entry or exit. Four of these openings are 0.17 m × 1.4 m, and form the eave spaces of the OBS. The other three openings are smaller, measuring 0.08 m × 1 m, and are at a height of 0.70 m midway from the basement. These smaller openings are fitted to the inside with upward facing cloth barriers joined to the wall at an angle of 45 degrees; to reduce the number of mosquitoes that may exit the OBS through these openings. Mosquitoes can enter or exit the structure through any of the openings except the sleeved user entry point which remains closed when the OBS is in use.

To trap mosquitoes, interception exit traps (made of ultraviolet-resistant netting on a wire frame) can be fitted on two of the eave openings (Fig. 1B). This way, mosquitoes are let in freely without restriction and are trapped only as they attempt to leave. These exit traps are not essential when the OBS is used solely as a contamination station even though they may still be required to sample and thus monitor the mosquitoes that visit the device. The entire inside of the device is lined with black cotton cloth except the floor which is covered with a plastic floor mat. Also, depending on how it is used (either as a trap, a contamination site or both), panels of fabric impregnated with a contaminant of choice can be suspended inside from a crossbar under the roof cover.

The whole structure is suspended on pedestals attached underneath the wooden basement. To prevent crawling predators and scavengers such as ants from climbing into it during experimentation, bowls of water are put under the pedestals. The portability of the device is improved by constructing it in kit format so that each section (the side panels, the roof and the wooden basement) can be detached and transferred from one place to another, allowing the OBS to be reconstructed onsite. Even without complete deconstruction, the super structure itself can be detached from the wooden basement and each part carried separately over short distances.

### The mosquito lure used in the OBS

The design of the OBS allows for different types of mosquito lures to be used. However, in this study, a synthetic blend of attractants recently developed by our research group was used [19]. This lure consists of iteratively determined optimal concentrations of aqueous ammonia, l-lactic acid and several other carboxylic acids, all dispensed by evaporation in a continuous plume of carbon dioxide. A batch of nylon strips soaked in the different constituents of the blend are hung inside a plastic pipe (30 cm length and 8 cm diameter), at the top of which there is a downward blowing fan driven by 6-volt battery. The carbon dioxide gas is introduced from a pressurised cylinder using rubber tubing fitted into the plastic pipe through a small hole on its side. This odor-dispensing method has previously been described in detail elsewhere [20].

### Study village

All the experiments described here were conducted in a malaria endemic rural village, Lupiro (8.385°S and 36.670°E), in Ulanga District, south eastern Tanzania. The village lies 300 meters above sea level on the flood plains of the Kilombero Valley and is approximately 30 km south of Ifakara town, where Ifakara branch of the Ifakara Health Institute (IHI) is located. Residents here experience perennially high malaria transmission and until recently, unprotected individuals could get as many as 352 infectious mosquito bites per year [21]. The village borders a permanent swamp extensively cleared for rice cultivation. Annual rainfall is approximately 1200-1800 mm while the temperature ranges between 20°C and 32.6°C. Malaria vectors in the area comprise primarily of the *Anopheles gambiae *complex, though there is also a small population of *Anopheles funestus*. A recent assessments have determined that 98% of sibling species within the *An. gambiae *complex in this area were *An. arabiensis*, the remaining being *An. gambiae sensu stricto *[19].

### Tests to optimize efficacy of the Ifakara OBS

The original design of the OBS had only four eave openings but did not have the three extra side openings as in the final design. Also, two of the eave spaces were originally fitted with barriers made of cotton fabric and slanting inwards and upwards at the same angle as the roof cover. It had originally been envisaged that these barriers would allow in mosquitoes but that they would restrict the exit of the mosquitoes through the same openings. During initial observations, it was determined in many occasions that there were several mosquitoes flying around the OBS especially near the eave spaces, but without going into the structure. Several of these mosquitoes were observed landing on the fabric barrier itself. It was hypothesised that these mosquitoes could not easily recognize the existing openings and that the fabric barrier on the eave space was actually restricting entry of some mosquitoes.

Therefore to improve efficacy, the design was modified by introducing the three additional openings on the sides (Figs. 1A and 1B) and removing the barriers from the eave spaces. To test whether these changes in design would increase the number of mosquitoes entering the device, field experiments were conducted in which the original intact OBS was compared to: 1) one with side openings added but the fabric barriers on eaves not removed and 2) one with the side openings added and the fabric barriers on the eaves removed.

The three OBS types (the different designs) were located in three different locations, 35 metres apart. They were fitted with interception exit traps on two opposite eave openings as shown in Fig. 1B, and the synthetic mosquito lure was dispensed from inside each of them. Each night, mosquitoes that entered the devices were sampled using the exit traps, sorted into different taxa and counted. The positions of the three designs were rotated so that at the end of each rotation, each OBS type had been to all the three locations. This experiment was replicated six times over 18 nights. The numbers of female mosquitoes caught in the exit traps was used to comparatively rank efficacies of the three designs.

### Test to evaluate trapping efficacy of the Ifakara OBS and to compare it with other outdoor mosquito traps

The trapping efficacy of the optimal Ifakara OBS design, when fitted with two exit traps (Fig. 1B), was compared to the trapping efficacies of two existing mosquito traps previously developed for use outside human dwellings. These other traps were: 1) an improved version of the Ifakara Tent trap, recently developed for sampling exophagic and endophagic mosquitoes [22,23] and 2) the Mosquito Magnet-X (MMX^®^), a counter flow geometry trap developed by American Biophysics Cooperation (ABC Ltd, North Kingstown R.I.) [24-26].

The experimental design was as follows: Three locations were identified in the study village, approximately 35 metres apart. In each location, the Ifakara OBS, the Ifakara Tent trap or the MMX^® ^were located. All the three traps were fitted with the same synthetic odor blend to lure mosquitoes [19,20]. Each night, the female mosquitoes caught in the different devices were sorted and counted. The positions of the traps were interchanged nightly so that at the end of the each complete 3-day rotation, each trap type had been to each location once. This experiment was replicated five times over 15 nights.

### Test to evaluate efficacy of the Ifakara OBS when used as a contamination station, and to compare different methods of dispensing contaminants

In the third experiment, the Ifakara OBS was evaluated as a contamination station rather than as a trap. The idea was that if all the openings are kept unblocked, a higher number of mosquitoes would enter the OBS than if some of the openings are blocked e.g. by fitting traps onto them. Whereas fitting traps onto the device enables one to catch and thus monitor mosquitoes that visit the device, the traps also reduce the available entry spaces for mosquitoes. It was hypothesised that perhaps this device would be more effective if most or all the attracted mosquitoes were let in and then contaminated so that they would die shortly after exiting the OBS.

To test whether the OBS could successfully be used as a contamination station, an experiment was conducted in which it was fitted with insecticide treated fabrics as follows: In one OBS, two panels of black cotton fabric, hand-treated by soaking in 1% aqueous solution of an organophosphate, pirimiphos-methyl, emulsified concentrate (Syngenta South Africa (Pty) Ltd. Midrand, South Africa), were hung from a cross-bar under the roof cover. In the second OBS, a similar set-up was installed but in this case the panel was made of white polyester netting as opposed to black cotton cloth. In both cases, the fabric panels were 1.5 m by 1.2 m in size. The different fabric materials were used so as to provide indications as to whether impregnation surfaces would affect the success of OBS contamination mechanism. This insecticide, pirimiphos-methyl was selected as the test contaminant because previous reports have suggested it as being toxic but having no repellent effects to mosquitoes [27], essential properties for this proof of principle experiment. Repellent insecticides would not be suitable for use in a lure and kill strategy, as they would reduce the number of mosquitoes entering the OBS and therefore the number of mosquitoes potentially affected by the insecticide or contaminant used.

All three OBS (one with insecticidal black cloth, one with insecticidal polyester net and the control) were baited using the same synthetic lure as in the experiment above. A 3 by 3 Latin square experimental design was used whereby the devices were rotated to each of the three locations. Each night female mosquitoes visiting and exiting the three OBS were sampled using exit traps fitted on two eave spaces (Fig. 1B). A clean white cotton sheet was spread on the floor of all the OBS so that any dead mosquitoes could also be seen and collected each morning. The experiment was replicated five times over 15 nights.

The dead mosquitoes were separated, sorted and counted, while live ones were maintained on 10% glucose solution inside a field insectary and monitored for 24 hours. The mean indoor temperatures in the insectary were 29.1°C ± 3.0 during the day and 26.7°C ± 2.3 at night, and mean relative humidity was 70.6% ± 17.9 in the day and 75.7% ± 13.7 at night. After 24 hours dead and live mosquitoes were sorted into different taxa and counted. This way, we observed both the immediate mortality and the delayed mortality of mosquitoes contaminated when visiting the different OBS.

### Mosquito identification

The *Anopheles *species were first distinguished morphologically from *Culex *and *Mansonia *species. A total of 300 *An. gambiae sensu lato *mosquitoes were randomly selected for further identification using ribosomal DNA-polymerase chain reaction [28]. To constitute this total sample, 100 females were selected during each of the three experiments. A similar sample of the *Mansonia *mosquitoes was also morphologically identified further, to determine constituent species. The *Culex *mosquitoes could not be readily identified to species level but previous research in the same area has determined that most of them were members of the *Culex pipiens *complex comprising approximately 80% *Cx. pipiens quinquefasciatus *and 20% *Cx. pipiens pipiens *[29].

### Statistical analysis

Data were analysed using SPSS version 15 (SPSS inc. Chicago). The mean mosquito catches per OBS per night were first calculated and compared using either bar graphs showing 95% confidence intervals or tables. Further analysis was conducted using General Linear Models (GLM) as follows: Mosquito catches were modelled as a function of independent factors depending on the experiment, each time treating 'day' as a random variable to reflect daily fluctuations in mosquito numbers. Also, due to the heterogeneity of the mosquito counts, the data were log transformed to make it amenable to assumptions of the standard normal distribution. We also performed *post hoc *analysis using Tukey's test to assess differences between individual independent variables. With regard to the second experiment where trap efficiencies were compared, correlation coefficients were also calculated to establish whether the trapping efficiencies changed with changing mosquito densities.

## Results

**Figure 2:**
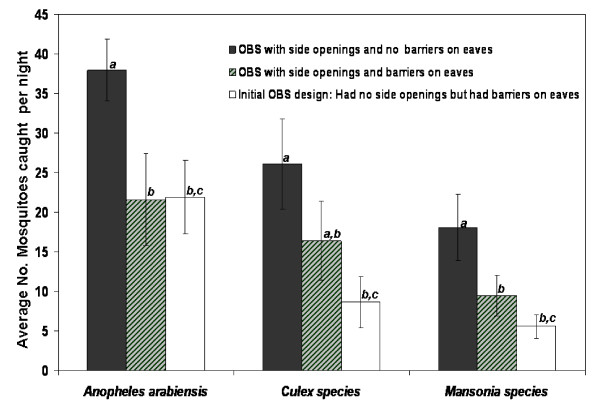
**Comparison of different designs of the OBS**. Average number of female mosquitoes caught per night inside the different OBS designs. The highest number of mosquitoes was caught when the OBS had side openings and no barrier on the eave spaces. The alphabetical symbols, **a, b **and **c **are used to represent differences as determined by Tukey's test. Trapping efficiencies are not significantly different (P = 0.05), if the different bars representing the designs share any of these alphabets. Designs sharing symbols are not significantly different The Y-error bars represent 95% confidence intervals.

In the first experiment, it was observed that the design of the OBS was a significant determinant of trapping efficacies against *An. arabiensis *(F = 8.279, P = 0.006, df = 2), *Culex *(F = 6.258, P = 0.027, df = 2) and *Mansonia *mosquitoes (F = 13.340, P = 0.001, df = 2). More mosquitoes were collected using the OBS with the side openings and no barriers on the eaves, than the other two OBS designs (Fig. 2). The actual differences between trapping efficacies of the individual designs as determined using Tukey's test are also shown in Fig. 2, using alphabetical symbols. Introducing side openings increased number of mosquitoes entering the OBS by between 0.99 and 1.89 fold, while introducing the openings and removing the eave barriers increased the number of mosquitoes by between 1.73 and 3.25 times. In this experiment, location did not affect catches of *An. arabiensis *(F = 0.264, P = 0.769, df = 2), *Culex *(F = 2.490, P = 0.093, df = 2) or *Mansonia *mosquitoes (F = 2.228, P = 0.118, df = 2).

Trap catches resulting from the experiment in which the Ifakara OBS was compared to the Ifakara Tent trap and the MMX^®^, are shown in Table 1. Trap type significantly affected the catches of *An. arabiensis *(F = 54.378, P < 0.001, df = 2), *An. funestus *(F = 4.979, P = 0.011, df = 2), other *Anopheles *mosquitoes (F = 114.662, P < 0.001, df = 2), *Culex *species (F = 61.207, P < 0.001, df = 2) and *Mansonia *species (F = 72.902, P < 0.001, df = 2). Both the OBS and the MMX^® ^were superior at trapping all mosquito species than the Ifakara Tent trap (Table 1). The *post hoc *analysis (using Tukey's test) showed that the number of *An. arabiensis *mosquitoes caught by OBS was not significantly different from MMX^® ^(P = 0.969), but the MMX^® ^caught significantly more *Culex *mosquitoes (P < 0.001) and more *Mansonia *mosquitoes (P < 0.001) than the OBS. Interestingly, even though most of the mosquitoes collected in the traps and the OBS were *Mansonia *and *Culex *species, the proportion constituted by the malaria vector, *An. arabiensis *was highest in the OBS catches (26.2%), followed by the Ifakara Tent trap catches (10.9%). This species constituted only 3.7% of the mosquitoes caught by the MMX^®^. As in the first experiment, location did not affect catches of *An. arabiensis *(F = 0.081, P = 0.922, df = 2), *An. funestus *(F = 0.791, P = 0.460, df = 2), other *Anopheles *species (F = 0.046, P = 0.956, df = 2), *Culex *(F = 1.138, P = 0.330, df = 2) or the *Mansonia *mosquitoes (F = 0.891, P = 0.418, df = 2).

**Table 1:** Comparison of the number of mosquitoes caught by the Ifakara Odor Baited Station (Ifakara OBS) when used as a trap versus the number of mosquitoes caught by the Mosquito Magnet-X (MMX^®^) trap and the Ifakara Tent trap♣

Trap	Anopheles arabiensis		Anopheles funestus		Other Anopheles species		Culex species		Mansonia species		Total No. Mos-quitoes
	**Mean** **(95%CI)**	**SUM** **(%)**	**Mean** **(95%CI)**	**SUM** **(%)**	**Mean** **(95%CI)**	**SUM** **(%)**	**Mean** **(95%CI)**	**SUM** **(%)**	**Mean** **(95%CI)**	**SUM** **(%)**	

MMX	37.2 (21.2-53.2)	558.0 (3.7)	4.3 (0.4-8.2)	64.0 (0.4)	29.6 (17.0-42.2)	444.0 (2.9)	685.9 (396.1-975.8)	10289.0 (67.5)	259.4 (183.6-335.2)	3891.0 (25.5)	15246

Ifakara OBS	34.1 (23.9-44.4)	512.0 (26.2)	1.7 (0.1-3.5)	25.0 (1.3)	0.9 (0.2.4)	14.0 (0.7)	73.4 (32.1-114.7)	1101.0 (56.3)	20.2 (12.1-28.3)	303.0 (15.5)	1955

Ifakara Tent trap	3.3 (1.4-5.3)	50.0 (10.9)	0.2 (0.0-0.4)	3.0 (0.7)	0.3 (0.1-0.5)	4.0 (0.9)	16.5 (11.4-21.6)	247.0 (54.1)	10.2 (5.6-14.8)	153.0 (33.5)	457

♣ The percentages are calculated on the basis of the total number of mosquitoes caught by any particular trap. For example in the case of the OBS, 26.2% of all the mosquitoes caught were *An. arabiensis*, while in the case of the MMX^®^, only 3.7% of all the mosquitoes caught in the trap were *An. arabiensis*.

Analysis performed to determine whether trap efficiencies depended on mosquito densities in any given night showed no statistical correlation between ratios of catches in the different traps and the average number of mosquitoes caught in each respective night. The daily ratio of OBS catches to MMX^® ^catches was not associated with the nightly mean catches (R^2 ^= 0.001, F = 0.001, df={1,13}, P = 0.992). Similarly the ratio of OBS catches to Ifakara Tent trap catches was not associated with respective nightly means of the catches (R^2 ^= 0.029, F = 0.011, df={1,13}, P = 0.919).

**Figure 3:**
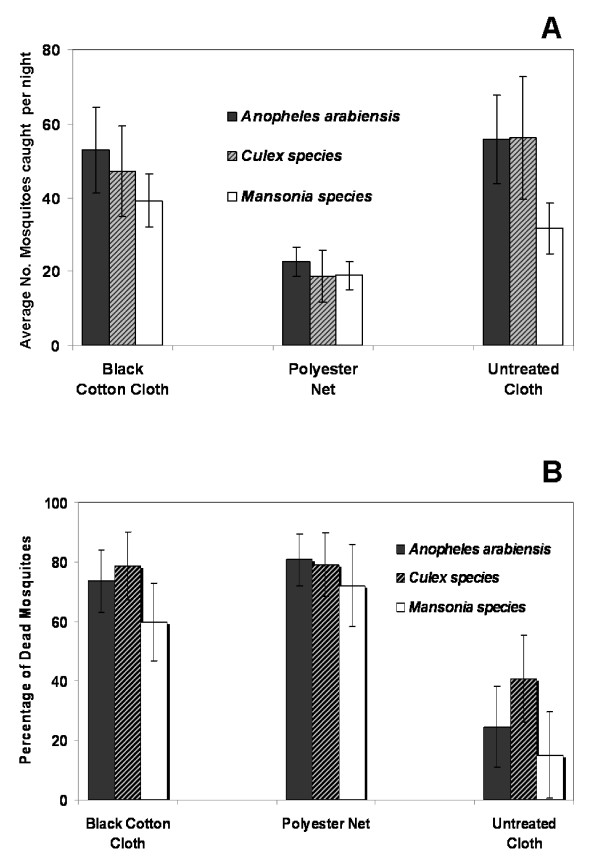
**Performance of the Ifakara OBS as a contamination station**. Panel **A): **Average number of female mosquitoes caught per night in the Ifakara OBS fitted with either polyester net or black cotton cloth treated with pirimiphos methyl. There were significantly less mosquitoes caught in the OBS with treated polyester net than with either the treated black cotton cloth or the control. Panel **B): **Percentage of all mosquitoes caught in the OBS that died within 24 hours. Significantly more mosquitoes died where the contaminant was used than in the controls. The Y-error bars represent 95% confidence intervals.

As shown in Fig. 3A, When insecticide treated fabric was introduced into the Ifakara OBS, the number of mosquitoes entering the devices was significantly affected by the type of fabric used, i.e. whether it was black cotton cloth, polyester netting or the control (F = 46.780, P < 0.001, df = 2). There were 59.3% fewer *An. arabiensis*, 66.8% fewer *Culex *and 40.21% fewer *Mansonia *mosquitoes in the OBS having polyester nets than in the control OBS. In this particular experiment, neither *An. funestus *nor any other *Anopheles *mosquitoes were caught.

Percentages of caught mosquitoes that died within 24 hours after exiting different OBS are shown in Fig. 3B. Using either fabric type, at least 73.6% *An. arabiensis*, 78.7% *Culex *mosquitoes and 60% *Mansonia *mosquitoes were contaminated and died within 24 hours. Unexpectedly, the mortality in the control were somewhat high, reaching 24.6% for *An. arabiensis*, 40.7% for *Culex *mosquitoes and 15.1% for *Mansonia *mosquitoes.

Ribosomal DNA analysis [30] of *An. gambiae *complex mosquitoes collected during the study showed that between 99% of the mosquitoes in this complex were *An. arabiensis*, only 1% were *An. gambiae s.s. *In this paper, the mosquitoes are therefore expressly referred to as *An. arabiensis *rather than *An. gambiae s.l. *On the other hand, the other *Anopheles *mosquitoes were 87% *An. coustani*, 11% *An. welcomi *and 2% *An. pharoensis*. Only two species of *Mansonia *mosquitoes were found namely, *M. africana *(67%) and *M. uniformis *(33%).

## Discussion

While historical evidence clearly suggests that vector control can be highly effective against mosquito-borne diseases [30-32], actual implementation of the strategy has not been optimal. Since a few years ago, there have been calls to restore the role of vector control by considering local evidence on disease ecology but also by developing new effective tools [7,33]; as existing methods may not be adequate to achieve relevant targets [9-11].

It has previously been suggested that synthetic odor cues that attract or repel mosquitoes could form the basis of new technologies for future control of mosquito-borne diseases [10,34], since these cues mediate important human-vector interactions that are associated with disease transmission [35]. For a long time, no chemical lure was known that could match or exceed attractiveness of natural human hosts, but recent research has led to one that is more attractive at long range than individual humans [19]. In their publication, the authors suggested a number of possible applications for this particular lure, of which odor-baited stations for trapping and killing mosquitoes as described here is the first field trial.

The technique of luring and killing vectors outside human dwellings has been used against other arthropods such as tsetse flies [36,37], but rarely for mosquito control. The current work reiterates extensive evidence that it is possible to lure mosquitoes to a predetermined location or structure outside human houses, but it goes further to show also, that other than simply trapping these mosquitoes as in many odor-baited traps [22,26,38,39], the mosquitoes can be killed or contaminated so that they continue to die even after leaving the target. The Ifakara OBS combines a simple trapping technology with a lure and kill strategy so as to maximise potential benefits for disease control. By letting in as many mosquitoes as possible with minimal restriction at entry, and by contaminating these mosquitoes, the overall efficacy of the OBS is greatly improved compared to what would be achieved by simply trapping the mosquitoes. In fact, if the OBS is used as a contamination station, it may be desirable that the embedded exit traps are removed so as to increase the area through which mosquitoes enter.

It is evident that this locally constructed device, though still relatively expensive (approximately USD 120 per piece), can attain trapping efficiencies that are better than or comparable to other existing outdoor traps. Moreover, whereas the other traps caught generally any mosquito species, the OBS was the one that was most specific for malaria vectors *An. arabiensis *and *An. funestus*; the proportion of catches constituted by these two vectors was higher for this device than for either Ifakara Tent trap or MMX^®^. In the original trials conducted inside experimental huts, where sleeping human volunteers were compared against the same synthetic lure used here, the lure attracted all mosquito species in similar proportions as real humans, leading to the conclusion that the sensitivity of the lure was not different from the sensitivity of natural human odors [19]. The differences observed in this current experiment therefore highlight the importance of trap designs on maximising capture rates of targeted important mosquito species.

The third experiment was primarily to establish whether Ifakara OBS could be used as a contamination station. However, it also compared two fabric types on the basis of suitability for dispensing the candidate contaminant, in this case pirimiphos-methyl. Polyester is commonly used in making insecticidal nets and was the first choice of material to use for our contaminant. On the other hand, black cotton cloth has been shown to encourage mosquito landing but also to effectively hold insecticides and biological agents [40,41].

Whereas both fabric types elicited high mortality on mosquitoes entering the OBS, it appears the treated polyester netting deterred mosquitoes from entering the OBS, while the black cotton cloth treated with the same insecticide did not. There may be different explanations for this observation: either the final insecticide concentration was higher on the polyester net and therefore was repellent rather than attractive to mosquitoes, or the mosquitoes preferred darker surfaces than the white polyester net. Nevertheless, since this was essentially a proof of principle experiment, perhaps the most important inference from the results is the need to carefully select appropriate dissemination medium for candidate contaminants, as this may significantly reduce or increase efficacies of the technology. The excess mortality observed in controls may have resulted from prolonged nearness to the synthetic odor lure, which consisted of 2.5% ammonia, 85% l-lactic acid and several other carboxylic acids. It is also possible that a few mosquitoes exiting the treated OBS later flew into the untreated OBS; a possible limitation of our study design. Besides, since we collected mosquitoes only once every morning, mosquitoes that had stayed in the exit traps the whole night may have been too exhausted to survive another day.

Though the lure and kill strategy was tested here using an organophosphate pirimiphos-methyl, other contaminants may also be used with the Ifakara OBS. Potential candidates include mosquito-killing fungi [42] and insect growth inhibitors like pyriproxyfen, which mosquitoes can transfer in small quantities back to their own aquatic breeding sites [43]. Similarly, though the Ifakara OBS was baited with only one synthetic lure [19], different other lures may be used as long as they are available, affordable and can fit inside the device. For example, where organic CO_2 _made from yeast and sugar [44,45] is used instead of the more expensive and hard-to-find industrial CO_2_, the usually bulky apparatus can simply be placed on the floor inside the OBS.

Finally, one exceedingly important question is how many such devices would be necessary for real life operational use in different epidemiological scenarios, where they should be located. Our previous work in which we used a mathematical model to estimate the potential benefits of odor-baited traps, suggests that where human settlements are clustered or where mosquito breeding grounds are identifiable, at least 20 of these devices would be required for every 1000 persons so as to match the efficacy of 50% coverage with long lasting insecticidal nets, but the optimal locations of the devices would need to be carefully identified, possibly by geostatistical modelling or participatory community mapping (Okumu et al Unpublished).

## Conclusion and recommendations

The Ifakara OBS is a potentially useful supplement to existing vector control tools, not only because it can be used to lure and trap disease-carrying mosquitoes but also because it can be used to contaminate and kill large numbers of mosquitoes that fly through it even if these mosquitoes eventually escape the embedded trapping mechanism. The OBS is easily constructed on site and can be baited with a variety of lures. Its portability can be improved if the wooden framework of the current design is changed so that the whole device assumes a cheaper, foldable and lighter tent format. Moreover, there remains the need to find cheaper, long lasting and more readily available mosquito lures, preferably not requiring industrial carbon dioxide gas as used in these experiments. Nevertheless, the work highlights possibilities to utilise synthetic lures in strategies for future vector control.

## Competing interests

The authors declare that they have no competing interests.

## Authors' contributions

FOO, DLW, RDS conceived the experiments. FOO, EPM, conducted the experiments, ANJ performed molecular identification of the mosquitoes and FOO drafted the original manuscript. All authors participated in writing the final draft of the manuscript prior to submission.
